# Adsorption of monoclonal antibody fragments at the water–oil interface: A coarse-grained molecular dynamics study

**DOI:** 10.1063/5.0207959

**Published:** 2024-06-25

**Authors:** Suman Saurabh, Li Lei, Zongyi Li, John M. Seddon, Jian R. Lu, Cavan Kalonia, Fernando Bresme

**Affiliations:** 1 Department of Chemistry, Molecular Sciences Research Hub, Imperial College, W12 0BZ London, United Kingdom; 2 Biological Physics Group, School of Physics and Astronomy, Faculty of Science and Engineering, Oxford Road, The University of Manchester, Manchester M13 9PL, United Kingdom; 3 Dosage Form Design and Development, BioPharmaceutical Development, BioPharmaceuticals R&D, AstraZeneca, Gaithersburg, Maryland 20878, USA

## Abstract

Monoclonal antibodies (mAbs) can undergo structural changes due to interaction with oil–water interfaces during storage. Such changes can lead to aggregation, resulting in a loss of therapeutic efficacy. Therefore, understanding the microscopic mechanism controlling mAb adsorption is crucial to developing strategies that can minimize the impact of interfaces on the therapeutic properties of mAbs. In this study, we used MARTINI coarse-grained molecular dynamics simulations to investigate the adsorption of the Fab and Fc domains of the monoclonal antibody COE3 at the oil–water interface. Our aim was to determine the regions on the protein surface that drive mAb adsorption. We also investigate the role of protein concentration on protein orientation and protrusion to the oil phase. While our structural analyses compare favorably with recent neutron reflectivity measurements, we observe some differences. Unlike the monolayer at the interface predicted by neutron reflectivity experiments, our simulations indicate the presence of a secondary diffused layer near the interface. We also find that under certain conditions, protein–oil interaction can lead to a considerable distortion in the protein structure, resulting in enhanced adsorption behavior.

## I. INTRODUCTION

Monoclonal antibodies (mAb) are therapeutic proteins that are gaining significant attention for their potential in treating cancer and infectious and autoimmune diseases.^1,2^ However, there are limitations associated with the stability of the formulated suspensions, which depend on the desired use and administration mode (intravenous or subcutaneous). Formulations for subcutaneous administration often involve significant mAb concentrations (>100 mg ml^−1^), and protein–protein interactions thus play an important role in determining the aggregation and stability of suspensions.

Previous studies have provided evidence for protein aggregation in bulk solutions. Non-native aggregation is a feasible degradation pathway for mAbs,^3^ with different buffers modulating the inter-protein interaction and suspension stability differently. The aggregation process is also influenced by mechanical agitation^4^ or temperature stress,^5^ in addition to aggregation-prone regions.^6^

Proteins can adsorb at interfaces. Indeed, mAb surface activity has been demonstrated in several experiments. In particular, Lu *et al.* performed a neutron reflectivity study of mAbs that showed surface adsorption of the monoclonal antibody COE3 and its isolated Fc and Fab fragments at both the air–water^7^ and water–oil^7,8^ interfaces. In the latter case, hexadecane was used to model the silicone oil/water interface in pre-filled syringes. Additional experiments using x-ray reflectivity^9^ of immunoglobulin G demonstrated antibody adsorption at the water–air interface. Moreover, these studies demonstrated that the mAb adsorption dynamics are very slow, and the dynamic surface tension features a long-time decay spanning from minutes to hours.

Protein adsorption and protein–interface interaction can modify protein structure and potentially drive in-plane aggregation at liquid interfaces. Leiske *et al.* provided evidence for protein unfolding at the water surface using fluorescence experiments.^10^ Experimental studies using synchrotron radiation circular dichroism experiments of small proteins (lysozyme, bovine serum albumin, and myoglobin) at the oil–water interface also revealed significant changes in the structure of the adsorbed proteins. The structural modifications included an *α*-helical structure reduction and an increase in *β*-sheet content.^11^

According to the analysis of previous experiments, protein adsorption is a time-dependent process that can induce the unfolding of proteins. The unfolding can lead to an increase in apparent protein hydrophobicity due to exposure of hydrophobic amino acids. Such changes in the protein structure can result in aggregation, which can be a significant challenge in designing and processing therapeutic formulations,^12^ and can affect protein therapeutic efficacy as well.^13^ While mechanical perturbations are known to trigger protein aggregation,^4^ the studies discussed above clearly indicate that the adsorption process might also induce aggregation in mechanically unperturbed samples.

The protein–interface interaction can be characterized using interfacial rheology.^14^ Shear stress measurements revealed that small proteins (BSA, lysozyme, and insulin) exhibited a viscoelastic response at the oil-water interface.^12^ More recently, Wood *et al.*^15^ conducted shear rheology experiments using the anti-streptavidin immunoglobulin-1 antibody.^15^ The results suggest that the viscoelasticity of the protein films is similar to that of a soft glass, and the proteins do not form gel-percolating networks.

We need more molecular insight to understand and predict the structural changes induced by interfaces on proteins. Specifically, the level of perturbation required to enhance mAb hydrophobicity and interfacial adsorption is unclear, namely, how extensive the local unfolding is and how many amino acids are involved in the process. Computer simulations can help us answer these questions by quantifying protein adsorption.^16^ Recently, molecular dynamics (MD) simulations were used to uncover the mechanism responsible for the stabilizing effect of histidine buffer,^17^ as well as the role of various buffers like citrate, in enabling inter-protein bridging.^18,19^ Moreover, MD simulations of small proteins, such as lysozyme, in combination with sum frequency generation, have provided insight into the protein conformation and orientation at the water–air interface. These simulations also offered insight into the dependence of protein orientation with the solution pH.^20^ Atomistic simulations of full monoclonal antibodies at the water–vapor interface have also contributed to understanding the mechanism behind interfacial adsorption.^21^ However, it is worth noting that the use of MD to simulate antibodies and their Fc and Fab fragments, especially at interfaces, is still in its early stages.

This work presents molecular dynamics simulations of the Fab and Fc fragments adsorbed at the oil–water interface. To do this, we use the MARTINI 3^22^ coarse-grained model, which allowed us to investigate multi-protein systems and the corresponding protein interfacial layers. Our multi-protein systems consist of as many as 176 coarse-grained proteins simulated for up to 2 *μ*s. With these simulations, we gain insight into the structural arrangement of the proteins and the structure of the adsorbed protein layers.

## II. RESULTS

### A. Reparameterization of the water–oil and protein–water interactions

Following earlier experiments,^23^ we performed simulations of the oil–water interface, using hexadecane to represent the oil phase. As noted in the experimental work, hexadecane provides a model that mimics the properties of the silicone oil/water interface. This interface is relevant in medical applications, and is, specifically, present in syringes containing mAb suspensions.

**FIG. 1.**
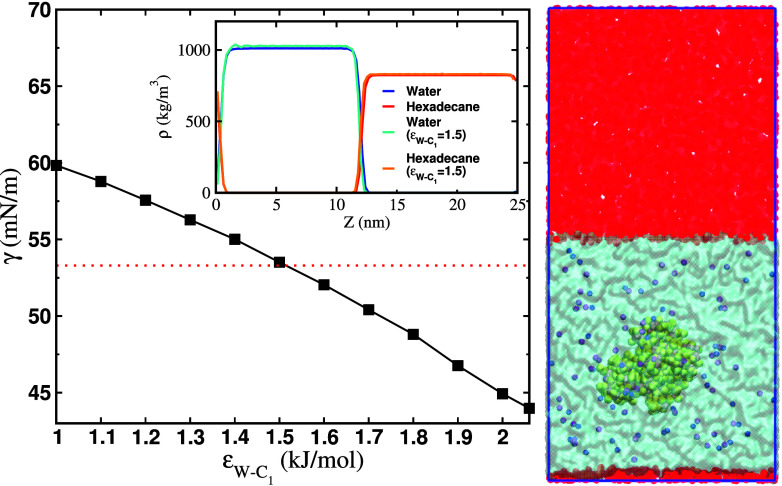
The interfacial tension of the water–oil interface as a function of the LJ interaction energy between the W–C_1_ pair. The red-dotted line represents the experimental value.^24,25^ The inset shows the liquid density profiles (for the original and modified MARTINI 3 parameters) as a function of the position along the direction perpendicular to the interface plane. The panel on the right shows the setup to simulate the protein–interface interaction. Initially, the protein was placed at the center of the water slab (shown in cyan). Hexadecane is shown in red, and the protein is colored in yellow. The Na^+^ and Cl^−^ ions are shown in blue and magenta, respectively.

The experimental interfacial tension (*γ*) of the hexadecane–water interface at 300 K and 1 bar pressure is 53.5 mN/m.^24,25^ We performed initial simulations using the original MARTINI 3 parameters under these thermodynamic conditions. We used a barostat in the direction normal to the interface plane, and the interface cross-sectional area was maintained constant. MARTINI 3 predicts an interfacial tension, γ= 44.5 ± 0.05 mN/m, which is lower than the experimental value. To reproduce the experimental result, we systematically reduced the strength of the LJ interaction (*ε*) between the W (water bead) and C_1_ beads [(C_1_)_4_ is hexadecane]. This change in the cross interaction does not modify the original water–water and hexadecane–hexadecane interactions, which are modeled using the original MARTINI 3 parameters. Figure 1 shows the variation of *γ* as a function of $\varepsilon_{W-C_{1}}$. The interaction parameter $\varepsilon_{W-C_{1}}$ = 1.5 kJ/mol gives γ= 53.3 ± 0.4 mN/m (for a short-range cut-off of 1.3 nm) and reproduces the experimental interfacial tension within the uncertainty of our computations.

As expected, the change in the water–oil cross interaction does not modify the bulk density of the two components (see inset of Fig. 1), *ρ_w_* = 1010 kg/m^3^ and *ρ_hd_* = 830 kg/m^3^, which are similar to the values obtained using original MARTINI 3 parameters and are comparable to the experimental values of 1000 and 770 kg/m^3^, respectively. All simulations presented below were performed using $\varepsilon_{W-C_{1}}$ = 1.5 kJ/mol.

**FIG. 2.**
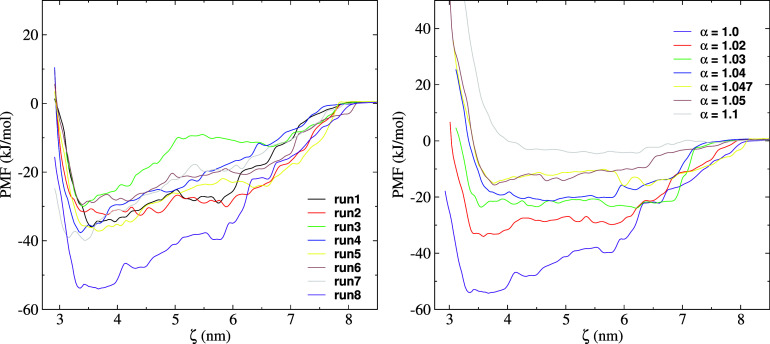
(Left) Fc–Fc PMF profiles obtained from eight independent runs with different initial relative orientations of the two Fc domains using the original MARTINI parameters (see Fig S3 in the supplementary material). (Right) PMF profiles for the different scaling factors used in this work.

An accurate description of the inter-protein interaction is important to model protein adsorption correctly. It has been noted that the MARTINI force field overestimates attractive interaction between proteins,^26^ and that it predicts conformations for disordered and multi-domain proteins in simulations that have enhanced compactness.^27^ To correct for this effect, the force field needs to be modified. The second virial coefficient (B_22_) provides a route to calibrate such interactions using experimental data as a reference. We calculated the B_22_ values for the Fc fragment from simulations and compared with the experimental value from our previous work.^28^ The values obtained from simulations did not match the experimental data. To address this, we rescaled systematically the water–protein vdW interaction in the MARTINI 3 force field, and identified the rescaling factor that results in B_22_ values in accordance with the experiments. Scaling up of the water–protein interaction leads to a more hydrophilic protein surface and reduces the tendency of the proteins to aggregate. B_22_ for the protein–protein interaction was computed from simulations by calculating the potential of mean force (PMF) (see Sec. IV). The Fc–Fc PMFs were calculated as a function of the inter-protein distance, using eight different trajectories (see Fig. 2). These runs correspond to eight different initial Fc-Fc relative orientations (see Fig. S3 in the supplementary material). All the PMF profiles predict strong attraction between the Fc proteins (see Fig. 2). The PMF profiles with error bars calculated using bootstrapping from the eight umbrella sampling simulations are shown in Fig. S5 of the supplementary material. Some trajectories lead to very strong attraction (>50 kJ/mol; see data for run 8 in Fig. 2). The PMF with the strongest attractive interaction is more likely in a situation where the fragments can explore the phase space sufficiently. Therefore, we adopted the orientation leading to the strongest attraction as representing the most likely orientation of the proteins at close contact and used the corresponding PMF profile to compute the second virial coefficient.

Starting from the conformation used for run 8, we rescaled all the protein–water LJ cross-interaction energy by factors (*α*) of 1.02, 1.03, 1.04, 1.047, 1.05, and 1.1. The protein–protein and water–water interactions were modeled with the original MARTINI 3 parameters. We calculated the PMF profiles using the rescaled parameters. The PMF profiles obtained for each value of *α* are shown in Fig. 2 (right). The PMF profiles obtained using the rescaled parameters along with error bars calculated using bootstrapping are shown in Fig. S6 of the supplementary material. These PMFs were used to calculate the second virial coefficient, *B*_22_, using Eq. (1). Our *B*_22_ (see Table I) values vary significantly with the cross-interaction parameter *α*. The original MARTINI 3 parameters overestimate the magnitude of *B*_22_ and, therefore, overestimate the inter-protein attraction. From interpolation of the data in Table I (see Figs. S7 and S8 in the supplementary material), we find that the parameter α=1.043 results in a virial coefficient in good agreement with the experiment.^28^ We tested the applicability of this parameter to other proteins by simulating lysozyme (PDB id. 1HEL) using the elastic network model. The values of B_22_ obtained for different values of *α* are listed in Table I. The scaling factor (∼1.045) required to reproduce the experimental B_22_ of lysozyme (at pH = 7 and NaCl concentration of 100 mM)^26^ is very close to the one obtained for the Fc fragment. We also find that our values for *α* are close to those considered in an earlier study of disordered and multi-domain proteins ( α= 1.1),^27^ using the MARTINI 3 force field.

**TABLE I.** Virial coefficient as a function of the rescaling factor for the Fc fragment of mAb COE3 and lysozyme.

	$B_{22}\times$ 10^−2^ (mol ml g^−2^)	$B_{22}\times$ 10^−5^ (mol ml g^−2^)
*α*	Fc	Lysozyme
1.0	−3 784 470 ± 1 015 232	−3386 ± 2409
1.02	−1998.6 ± 206.4	−1523.7 ± 574
1.03	−127.2 ± 11.3	−469.5 ± 134
1.04	−35.5 ± 4.6	−85.7 ± 41.4
1.047	−4.72 ± 0.5	+84.5 ± 17.2
1.05	−1.85 ± 0.17	+91.5 ± 20.9
1.1	−0.045 ± 0.012	+156.8 ± 16.6
Experiment	−7.77 ± 1.55 (from Ref. 28)	−30 (from Ref. 26)

Rescaling the protein–water interaction might influence the amino acids' preference for the oil and water phases, making the amino acids more soluble in the water phase. We computed the free energy difference of the hydrophobic amino acids (according to the Black and Mold scale^29^) in the aqueous and oil phases, using the original and rescaled protein–water interaction parameters to estimate the effect of rescaling on the solubility of amino acids.

**FIG. 3.**
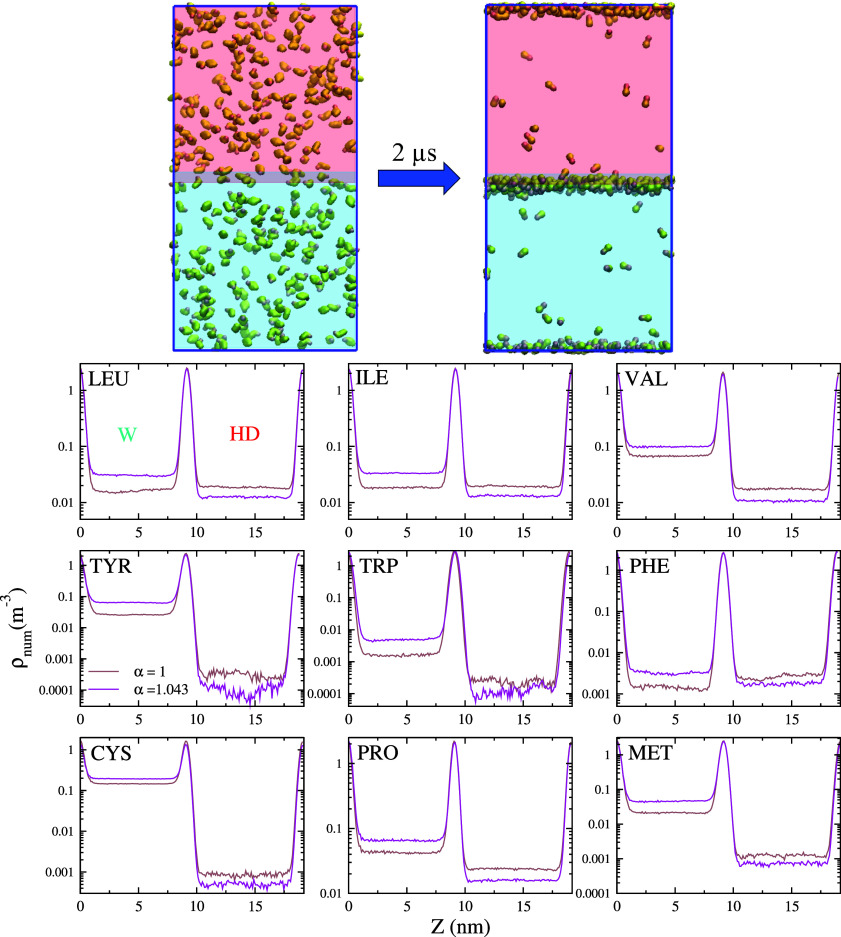
Simulation setup employed to compute the free energy difference of the amino acids in the oil and water phases. Left and right panels represent the initial and final configurations after a 2 *μ*s simulation. The lower panels represent the number density for each amino acid type. The results were obtained using both the original MARTINI 3 parameters and the rescaled ones proposed in this work. The side chains of all the amino acids were represented by the respective MARTINI 3 beads, while the neutral MARTINI 3 P2 bead represented the backbone.

A typical simulation for the free energy calculations consisted of 340 amino acids in a simulation box with dimensions 10 × 10 × 20 nm^3^ containing 2200 hexadecane and 7800 water beads. The number of amino acid beads is large enough to obtain good statistics and sufficiently small to prevent significant interaction between amino acids for the given box volume. The amino acid to water mole fraction is ∼0.01. As noted earlier, for this mole fraction, the results approach that of the infinite dilution system.^30^ The upper half of the simulation box (along the Z-direction) was filled with hexadecane, while the lower half was filled with water (see Fig. 3). For each amino acid species, three independent simulations were run for 2 *μ*s. During the simulation, the amino acids partition into the bulk water and hexadecane phases. The number density profiles of the amino acids along the Z-direction (see Fig. 3) show well-defined plateaus in the water and oil phases and adsorption maxima at the oil–water interface. The number densities are calculated as the density of the centers of mass of the amino acids and are thus independent of the number of beads constituting the amino acid. A higher number density in oil relative to the water phase indicates that the corresponding amino acid is more hydrophobic. For instance, Leu, Ile, and Phe feature the highest number densities in hexadecane. As expected, for the rescaled parameters, the density in the oil phase is lower than that for the original parameters. We calculate the free energy difference of the amino acids in the water or hexadecane phases as

$$\Delta G^{HD/W}=k_{B}T\;\text{ln}\;\left(\frac{\rho^{HD}}{\rho^{W}}\right),$$ (1)

where *ρ^HD^* and *ρ^W^* are the number densities in the oil and water phases, respectively. The free energy differences for amino acids with hydrophobic side chains for the original and rescaled MARTINI parameters are shown in Table II. The relative hydrophobicity of the different amino acids is preserved to a large extent when we use the modified *α* parameter. The simulations predict the same free energy difference for Leu, while Pro and Val feature a 25% change. Hence, the difference in free energies due to rescaling depends on the amino acid species. The impact of changes in the free energy difference on protein adsorption will depend on the amino acid composition at the protein surface. A large number of amino acids associated with a significant change in free energy difference upon rescaling may lead to a substantial difference in the adsorption behavior of the protein, and balancing of interactions through rescaling of protein–oil interactions would need to be employed. Advancing the discussion later, we note that the change in free energy differences, due to the rescaling, results in small changes in the orientation of the Fab fragment at the water–hexadecane interface and almost no change in that of the Fc fragment.

**TABLE II.** Free energy differences, $\Delta G^{HD/W}/kT$, of hydrophobic amino acids for the original and rescaled MARTINI parameters.

	Free energy difference	Free energy difference
Amino acid	(*α* = 1)	(*α* = 1.043)
LEU	−0.89 ± 0.03	−0.86 ± 0.05
ILE	−0.84 ± 0.10	−1.04 ± 0.10
VAL	−1.21 ± 0.05	−1.52 ± 0.10
TYR	−1.02 ± 0.03	−1.05 ± 0.02
TRP	−0.88 ± 0.12	−0.98 ± 0.20
PHE	−0.72 ± 0.11	−0.84 ± 0.07
CYS	−1.72 ± 0.03	−2.04 ± 0.05
PRO	−1.00 ± 0.15	−1.24 ± 0.02
MET	−1.14 ± 0.06	−1.28 ± 0.05

Unless otherwise stated, the results below have been obtained with the rescaled MARTINI parameters.

### B. Surface activity of the Fc and Fab proteins at low bulk concentration

#### 1. Adsorption behavior and “hot spots” driving adsorption

We performed simulations targeting highly diluted protein suspensions, using a single protein interacting with the oil–water interface.

**FIG. 4.**
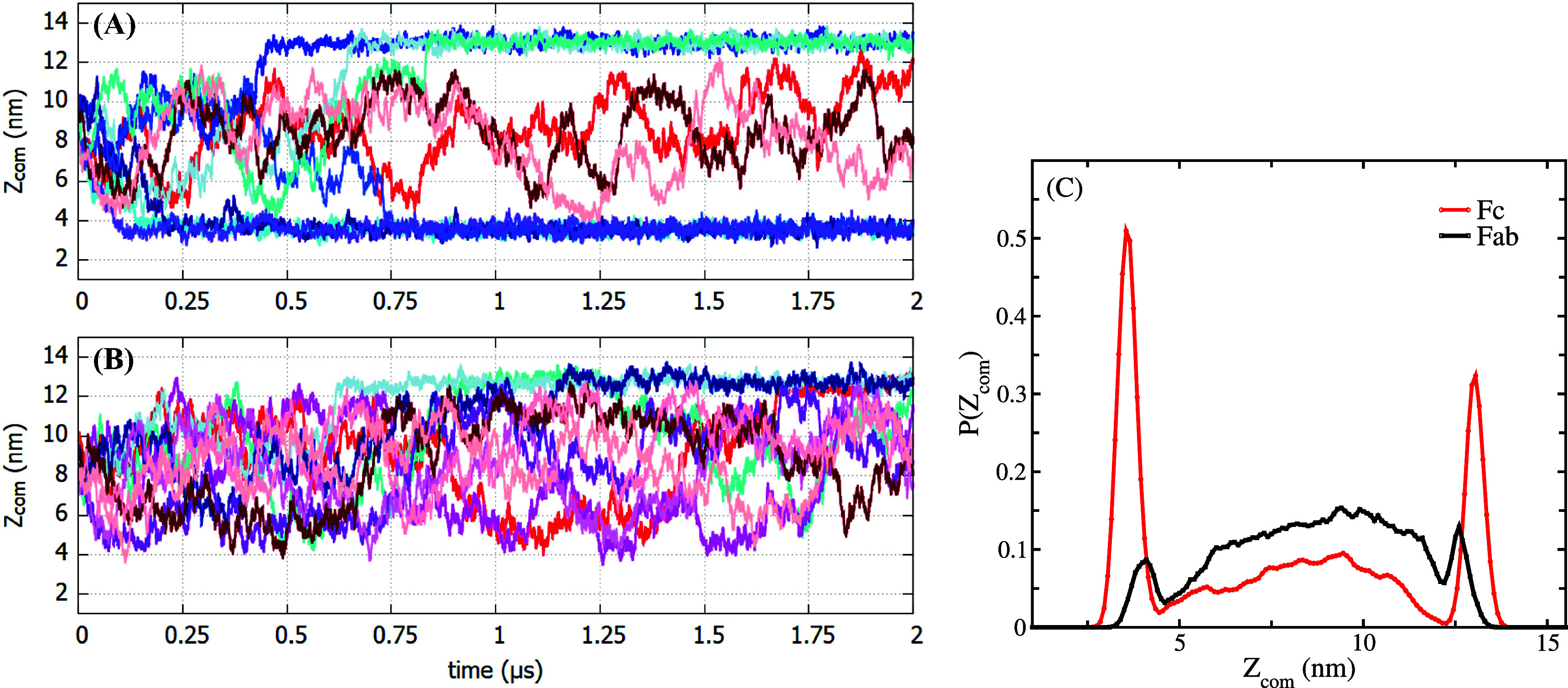
Z-coordinate of the center of mass (Z_*com*_) of the (a) Fc and (b) Fab fragments as a function of time for ten independent 2 *μ*s long simulations. (c) Normalized probability distribution of Z_*com*_ for the Fab and Fc fragments.

Figure 4 shows the position of the center of mass of the Fc and Fab fragments for ten independent simulations. The probability distributions show two prominent interfacial maxima for Fc [see Fig. 4(c)], indicating strong surface activity. The probability distribution for Fab features interfacial maxima too, albeit significantly smaller, indicating that this protein is less hydrophobic than Fc. This higher apparent hydrophobicity of the Fc fragment agrees with previous neutron reflectivity experiments.^23^

Figure 4(a) illustrates the differences in the adsorption behavior of the Fab and Fc fragments. The Fc fragment shows adsorption at the interface in 7 [shown in different shades of blue in Fig. 4(a)] out of the 10 trajectories investigated, whereas the Fab fragment shows persistent adsorption only in 3 out of 10 trajectories. Once the Fc fragment gets trapped at the interface, it stays there for a long time, whereas the Fab fragment features multiple short encounters with the interface, and is reflected back into the aqueous phase, owing to the lower hydrophobicity of the Fab surface. The relatively higher reversibility of the adsorption process for the Fab fragment, namely, the higher affinity to attach or detach from the interface, is a reflection of the lower hydrophobicity of Fab, and this explains why, in experiments, the same bulk concentration of Fab and Fc leads to a higher interfacial density of Fc compared to Fab.^23^

To shed light on the different adsorption behaviors of the Fc and Fab fragments, we have identified the protein surface hot spots for adsorption. To do this, we computed the total protein adsorbed area (A_*ads*_), and the contributions to it from the individual amino acids.

The total area of the protein adsorbed at the interface decreases with increasing protein–water interaction (i.e., increasing *α*). The average area for the Fab fragment reduces from $A_{\text{ads}}=$ 7.1 ± 2.5 nm^2^ (*α* = 1) to 3.2 ± 1.3 nm^2^ ( α=1.043). For the Fc fragment, we observe a smaller decrease, from $A_{\text{ads}}=$ 9.4 ± 1.7 nm^2^ (*α* = 1) to 7.8 ± 1.5 nm^2^ ( α=1.043). This reduction is consistent with the slight decrease in the amino acid hydrophobicity associated with stronger protein–water interaction (see Table II).

**FIG. 5.**
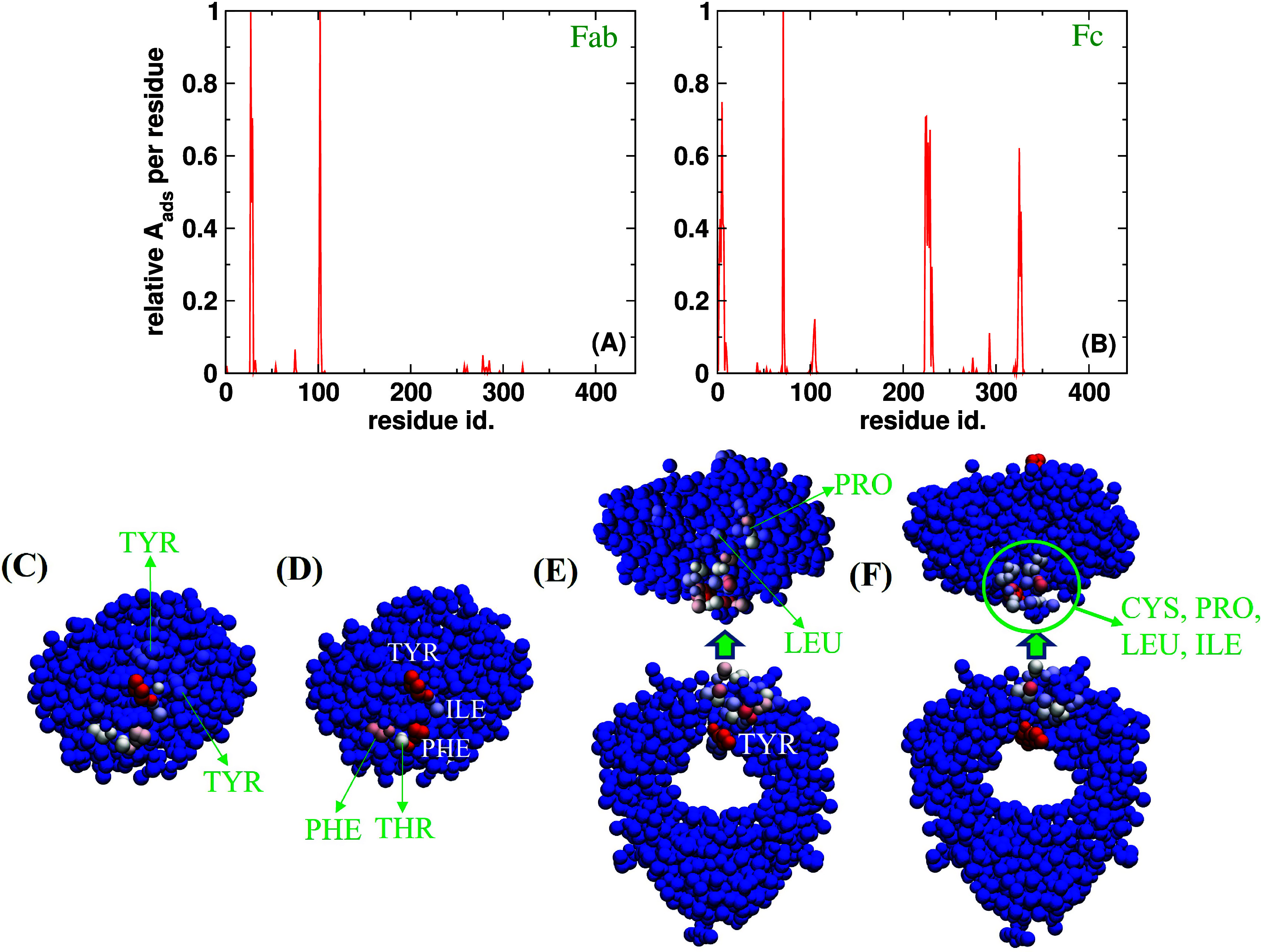
Adsorbed area (A_*ads*_) per residue for (a) Fab and (b) Fc fragments for the rescaled MARTINI 3 parameters, divided by the respective maximum *A_ads_* values, 0.15 and 0.42 nm^2^ for Fab and Fc, respectively. The surface active regions for Fab (c) and (d) and Fc (e) and (f) are colored according to their A_*ads*_. The plots show results for the original (c) and (e) and rescaled (d) and (f) parameters.

We also analyzed the individual amino acid contributions to the total surface area to identify surface active regions on the protein surface. Figure 3 shows the ${\left(A_{\text{ads}}\right)}_{i}$ of each amino acid *i* in the Fab and Fc fragment, averaged over the ten independent trajectories. In this relative scale, the ${\left(A_{\text{ads}}\right)}_{i}$ of the amino acid with the highest protrusion into the oil phase is equal to 1. Figure 3 shows that a small number of amino acids contribute significantly to the surface activity. Moreover, rescaling the protein–water interaction using the parameter *α* leads to minor changes in the surface active regions. Figures 5(c)–5(f) shows the location of the surface active amino acids. The location of the surface active hot spots is more or less independent of the interaction parameter (*α*). However the contribution of the amino acids to protein adsorption varies slightly with the interaction parameter. Figure 5(e), for instance, shows that the region around the Pro residue contributes much less to the surface activity for the rescaled parameters. The snapshots in Figs. 5(c)–5(f) indicate a strong preference for the protein to adsorb through a small region, a hotspot, on the protein surface. These hot spots contain typical hydrophobic amino acids, Leu, Pro, Phe, Thr, and Tyr (see also Table II), and will lead to a characteristic orientation when the protein interacts with the oil–water interface. We analyze the orientation in Sec. II B 2.

#### 2. Evidence for preferred protein–interface interactions

**FIG. 6.**
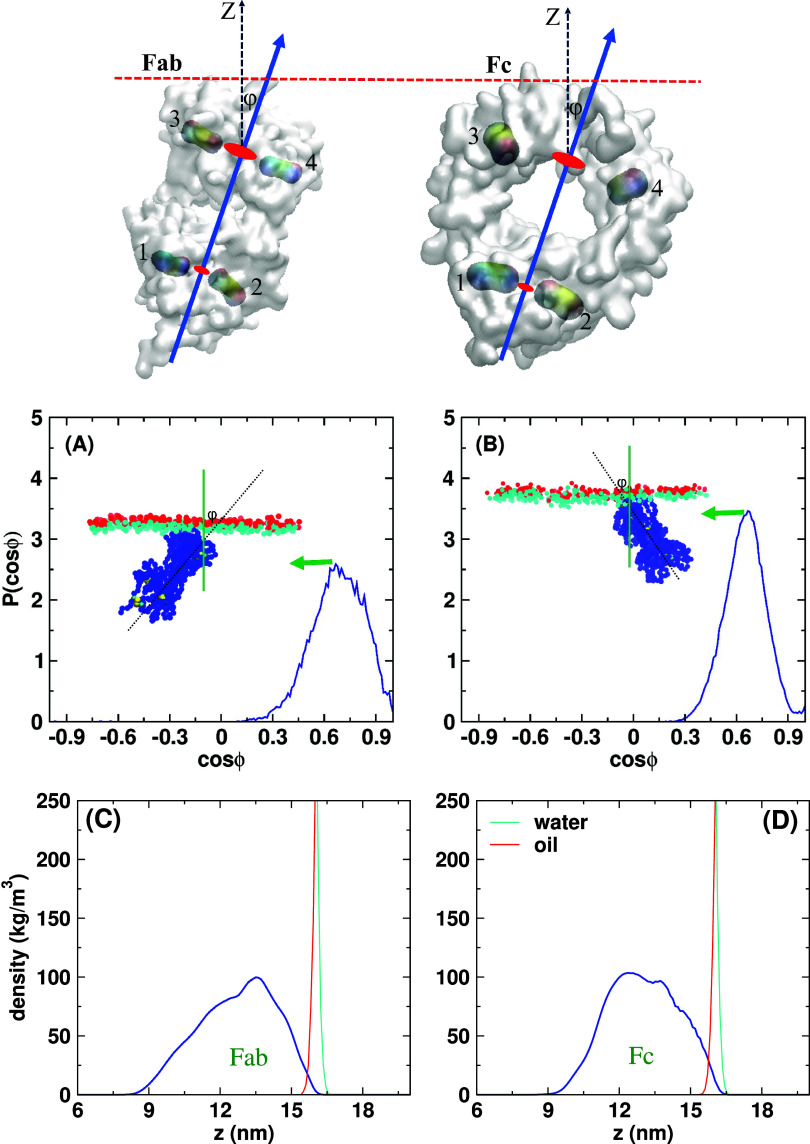
(Top panel) The disulfide bond pairs (1,2) and (3,4) defining the fragment axis. The orientation of the (a) Fab and (b) Fc fragments at the water–oil interface, measured as the cosine of the angle between the fragment axis and the Z axis, for the rescaled MARTINI 3 parameters. The red, cyan, and blue spheres in the insets correspond to the water and oil beads at the interface, and the protein, respectively. Panels (c) and (d) show the density profiles along the direction perpendicular to the interface plane, averaged over sections of trajectories during which the protein adsorbs continuously at the interface for at least 0.5 ns. Red, cyan, and blue lines represent the density profiles of oil, water, and protein, respectively.

We analyzed the impact of the surface active hot spots on the protein orientation at the interface. The orientation ( ϕ) of the Fab and Fc fragments was defined using the angle between the vector along the Z-direction, normal to the interface plane, and the vector joining the center of mass of the cysteines forming the disulfide bonds (1,2) with the center of mass of the cysteines forming the disulfide bonds (3,4) (see Fig. 5). The probability distribution of cos ϕ is shown in Figs. 6(a) and 6(b).

A comparison between the distribution of cos ϕ for the original and rescaled parameters is presented in Fig. S9 in the supplementary material. The orientation of the Fc fragment does not depend significantly on the interaction parameter (*α*), with a value of 48° for both the original and rescaled parameters. In contrast, the results for the Fab fragment show a larger dependence, with the peak value shifting from 23° (*α* = 1) to 49° ( α=1.043). The amount of change in the orientation angles (∼0°–25°) indicates that the interaction parameter does not substantially alter the location of the Fab and Fc adsorption hot spots. The active sites for adsorption for both Fab and Fc fragments involve hydrophobic residues like Leu, Ile, Tyr, Phe, Cys, and Pro. Our probability distribution shows a dominant orientation for both Fab and Fc, with Fab featuring a larger orientational disorder. We show in Figs. 6(c) and 6(d), the protein density profiles projected along the *Z* axis, obtained by averaging over sections of the MD trajectories where the proteins adsorb continuously at the interface for at least 0.5 ns. These profiles also show a larger orientational freedom of Fab compared to Fc. Furthermore, they show a small degree of penetration of the proteins in the oil phase. The definition of penetration into the oil phase, the numerical values, and the comparison with experiments are discussed later. The density profiles for each adsorption event (lasting more than 0.5 ns) for the rescaled and original MARTINI parameters are shown in Fig. S10 in the supplementary material. Figure S10 also compares the average density profiles obtained with the rescaled and original MARTINI parameters. These results confirm that rescaling of the protein–water interaction has only a minor effect on the general adsorption behavior of the Fc and Fab fragments.

### C. Protein–interface interactions at high protein concentrations

Therapeutic formulations often involve high protein concentrations exceeding, in some cases, 100 mg/ml. We performed large-scale simulations of multi-protein systems to investigate the impact of protein concentration on adsorption. We performed the simulation of large systems consisting of 44 and 176 proteins placed close to the water–oil interface in a simulation box with cross sections of 25 × 25 and 100 × 100 nm^2^, respectively. The interfacial density of proteins in our simulations corresponds to a value of 30 nmol/m^2^, obtained in experiments studying interfacial adsorption.^23^ The different simulation box sizes were used to ensure proper modeling of the protein–protein interaction and to minimize finite-size effects.

**FIG. 7.**
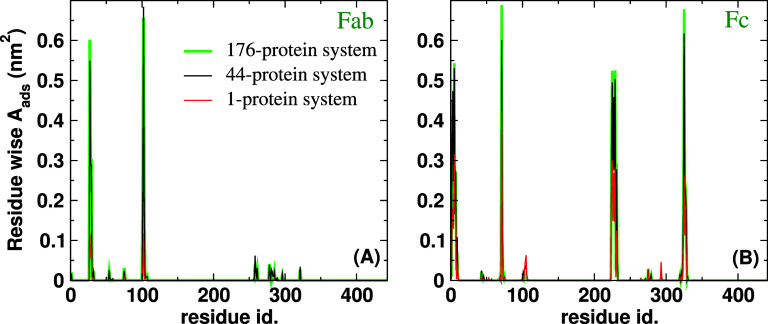
Residue-wise adsorbed area at the oil–water interface for single and multi-protein systems. Panels (a) and (b) represent results for the Fab and Fc fragments, respectively.

To understand the effect of a dense protein environment on protein adsorption at the water–oil interface, we calculated the residue-wise contribution to the total protein adsorbed area, A_*ads*_. This was done by first calculating the residue-wise A_*ads*_ for each protein, averaged over time. The time-averaged A_*ads*_ was then averaged for each residue over all proteins in the system. Furthermore, we performed an additional average over values obtained from three independent simulations. The results from this analysis are shown in Fig. 7. Our results demonstrate that the same residues adsorb at the interface for the single and multiple-protein systems. However, the value of A_*ads*_ for almost all surface active amino acids is larger for the multi-protein systems than for the single-protein system. The result indicates that the inter-protein interactions promote co-operative protein adsorption, i.e., the proteins co-adsorb.

We have further investigated the surface active regions in the multi-protein systems and identified the surface active amino acid sequences (consisting typically of 2–8 amino acids). We summarize this information for Fab and Fc in Table III. All these sequences contain at least one hydrophobic amino acid. Amino acids with hydrophilic side chains (like Thr and Asn) also adsorb at the interface due to their proximity to hydrophobic amino acids. For the Fc fragment, the hinge region contributes most of the surface active residues.

**TABLE III.** Amino acid sequences at the water–oil interface for the Fab and Fc fragments.

Protein	Adsorbing sequences
Fab
1.	Phe Thr Phe
2.	Ile Tyr Gly
Fc
1.	Tyr Asn
2.	Ala Leu Pro Ala Pro
3.	Pro Pro Cys Pro Ala Pro Gly Leu (Hinge)

**FIG. 8.**
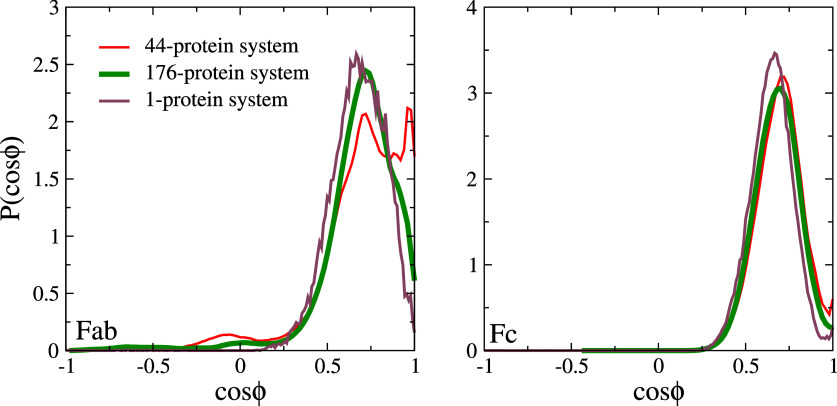
Orientational probability distribution of surface active proteins for the multi-protein simulations. (Left panel) Fab and (right panel) Fc. The data for the single-protein case (1-protein) are also shown.

We also investigated the orientational distribution of the proteins at the interface for the multi-protein systems and compared them with the single-protein case. We plot the distributions in Fig. 8. The larger orientational freedom observed in the single Fab simulations is reproduced in the multiprotein system (cf. Fig. 8). The impact of protein concentration is relatively minor, especially for the Fc fragment, where we find the peak and width of the distributions to be similar for the single and multi-protein systems. We observe some additional orientations for the Fab fragment in the multi-protein systems near cos ϕ = 0, but these have very low probabilities compared with the main peak. Overall, the effect of the protein–protein interaction in monolayers appears to be minor on protein orientation, but co-adsorption affected by inter-protein interaction leads to a change in the reversibility of adsorption, as reflected in the higher average residue-wise A_*ads*_ of the amino acids driving adsorption in the multi-protein case.

**FIG. 9.**
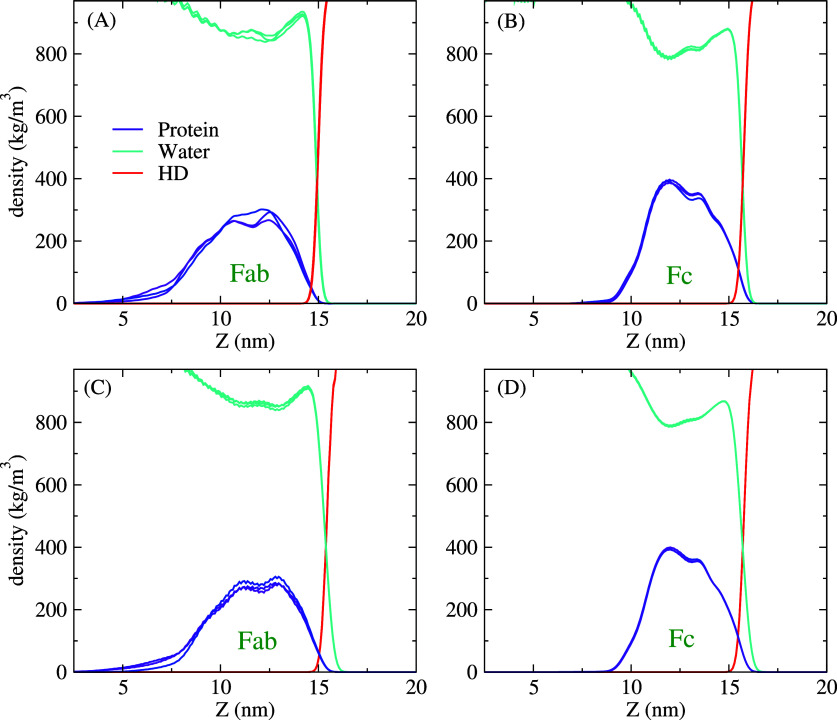
Protein, water, and hexadecane density profiles along the Z-direction (normal to the interface) for the three independent runs of the 44-protein (a) Fab and (b) Fc systems and the 176-protein (c) Fab and (d) Fc systems. The protein density profiles feature a slow decay toward the interior of the aqueous phase reaching zero eventually, suggesting that the adsorbed layers are in equilibrium with a bulk suspension with a very low protein concentration.

**FIG. 10.**
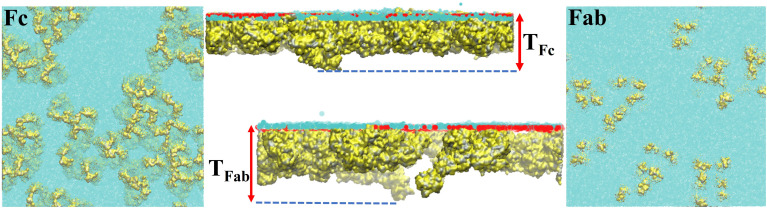
Top view of the structure of the protein layer at the water–oil interface for the Fc (left panel) and Fab (right panel) fragments for the 44-protein system. The oil phase has been removed to show the protrusion of protein (yellow) outside the water phase (cyan). The middle panel shows a lateral view of the protein layer (yellow). Cyan and red spheres show the water and oil molecules within 0.5 nm of the interface. The T_*Fc*/*Fab*_ refers to the thickness of the Fc/Fab layer.

In Fig. 9, we show the density profiles of the protein, oil, and water for the multi-protein systems. The density profiles are similar for both system sizes investigated in this work (44 and 176 proteins), presenting little dependence on system size. The Fab and Fc profiles show well-defined maxima, and a long tail extending into the water phase is visible in the case of the Fab. This tail emerges from proteins that are loosely connected to the adsorbed protein monolayer. Figure 10 illustrates the different adsorption behaviors of the Fab and Fc proteins. Fc occupies a higher surface area at the interface than Fab (cf. left and right panels in Fig. 10), as shown by the larger protrusion of the Fc proteins outside the water phase. Figure 10 also indicates that the Fc proteins form a more compact adsorbed layer (see the middle panel). For Fab, we observe dangling proteins attached weakly to the adsorbed Fab layer. These dangling proteins contribute to the tail shown in the density profiles represented in Fig. 8(c).

**FIG. 11.**
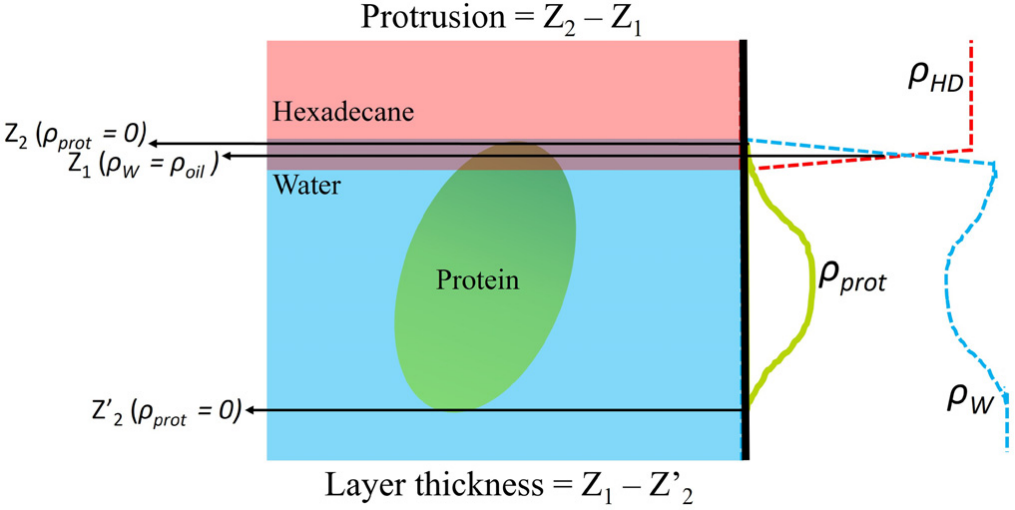
Sketch of the protein–water–oil interface illustrating our definition of protein protrusion into the oil phase and the layer thickness.

The protein surface activity can be characterized further by quantifying its protrusion into the oil phase. We define the protrusion as the difference between the Z-coordinate where the protein density at the interface reaches zero [z(*ρ_prot_* = 0)] and the Z-coordinate where the water and oil densities are equal [z(*ρ_oil_* = *ρ_wat_*), see Fig. 11]. z(*ρ_oil_* = *ρ_wat_*) was calculated from a linear interpolation of the function $\rho_{\text{wat}}(z)-\rho_{\text{oil}}(z)$ (obtained from the density profiles), to find the *z* coordinate at which this function is zero, i.e., the intersection point for both densities. We calculated the protrusion for the single and multi-protein systems (see Table IV). The simulations indicate that Fc experiences a stronger protrusion than the Fab fragment, supporting our earlier finding that the Fc domain is more hydrophobic than Fab. The protrusion in the case of the multi-protein systems is also slightly enhanced relative to the single-protein result, albeit the level of protrusion in all cases is small, <1 nm. This small value is consistent with the neutron reflectivity results,^23^ which used models to fit the reflectivity profiles. The protrusion inferred from the experiments is also small, of the order of the water molecular diameter. The neutron reflectivity results also showed similar levels of protrusion for the Fab and Fc fragments, a result that agrees with our simulations (see Fig. 10).

**TABLE IV.** Protrusion of the proteins into the oil phase and thickness of the adsorbed layer, obtained from simulations and reported in experiments. The experimental values correspond to the parameters used to fit the reflectivity profiles using the slab model discussed in Ref. 23.

	Protrusion into oil phase	Experiment	Layer thickness	Experiment
System	(nm)	(nm)	(nm)	(nm)
Fab (dilute system)	0.56 ± 0.003		8.36 ± 0.05	
Fab (30 nmol/m^2^)		0.35 ± 0.45 (Ref. 35)		5.9 (Ref. 23)
a) 44 proteins	0.68 ± 0.02		13.7 ± 0.2	
b) 176 proteins	0.71 ± 0.005		13.7 ± 1.1	
Fc (dilute system)	0.82 ± 0.002		8.6 ± 0.03	
Fc (30 nmol/m^2^)		0.2 ± 0.3 (Ref. 35)		6.4 (Ref. 23)
a) 44 proteins	0.88 ± 0.04		8.9 ± 0.8	
b) 176 proteins	0.97 ± 0.03		8.9 ± 1.1	

The thickness of the adsorbed Fab protein (see Fig. 11 for definition) increases from the diluted to the concentrated monolayer. This effect is connected mainly to the build-up of a weakly adsorbed secondary protein layer below the adsorbed protein monolayer (see Figs. 9 and 10). However, the thickness of the Fc layer is very similar for both diluted and multi-protein systems. This result is consistent with the more compact layer observed in the Fc case (see Fig. 10). The thickness of the Fc layer is similar to the estimated length of the Fc long axis, ∼8 nm.^28^

**FIG. 12.**
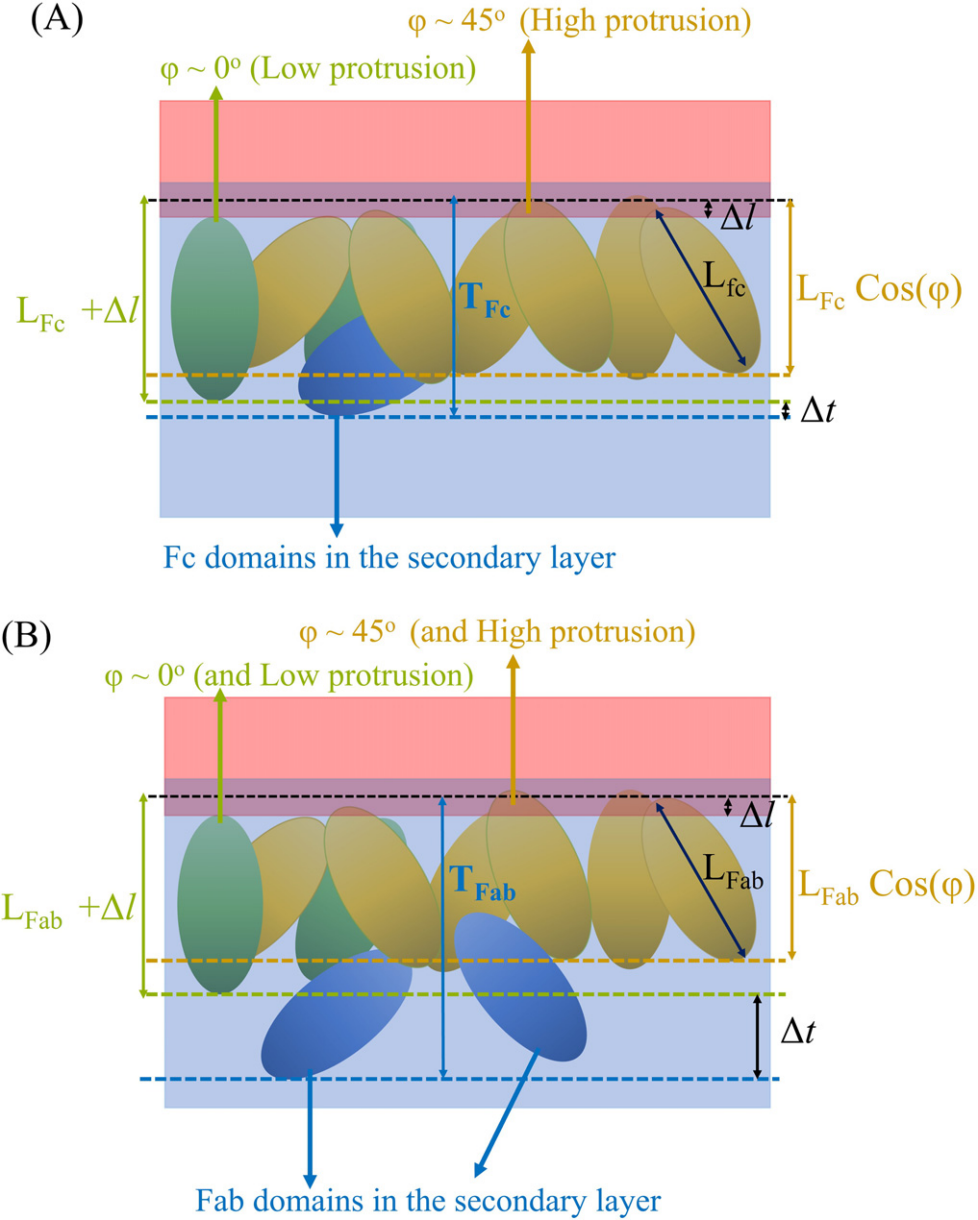
Cartoons illustrating the adsorbed layers of the (a) Fc and (b) Fab fragments at the water–oil interface. The water and oil phases are shown in blue and red, respectively. Proteins adsorbing with different orientations and protrusions are shown in different colors (see main text) .

Figure 12 shows cartoons illustrating the structure of the adsorbed protein layers at the water–oil interface. The reference line (shown in black in Fig. 12) identifies the location of the interface, namely, $\rho_{\text{wat}}(z)$ = $\rho_{\text{oil}}(z)$. The analysis of our simulation snapshots indicates that most of the protein fragments adsorb with orientations close to those corresponding to the maximum in the cos ϕ probability distributions (see Fig. 7), and protrude slightly above the interface plane. The thickness of the protein layer is, therefore, $L_{Fc/\text{Fab}}\cdot \;\text{cos}\;\phi$ (orange line on Fig. 12) with an uncertainty of Δl/2 (see Fig. 12 for a definition of Δl). The probability distribution of cos ϕ also shows that some fragments adsorb with an orientation ϕ = 0°. If such proteins have a low protrusion (green proteins in Fig. 12), they contribute to increasing the layer thickness beyond $L_{Fc/\text{Fab}}\cdot \;\text{cos}\;\phi$ to a value close to $L_{Fc/\text{Fab}}$ + Δl. A secondary layer consisting of the fragments (blue fragments in Fig. 12) that do not lie at the interface but form contacts with the fragments in the primary protein layer further increases the layer thickness, leading to a layer thickness that is larger than the overall length of the protein long axis. This effect is significant for the Fab fragment, and is connected to proteins loosely holding onto the main adsorbed layer [see proteins colored in blue in Fig. 12(b) and snapshots in Fig. 10].

**FIG. 13.**
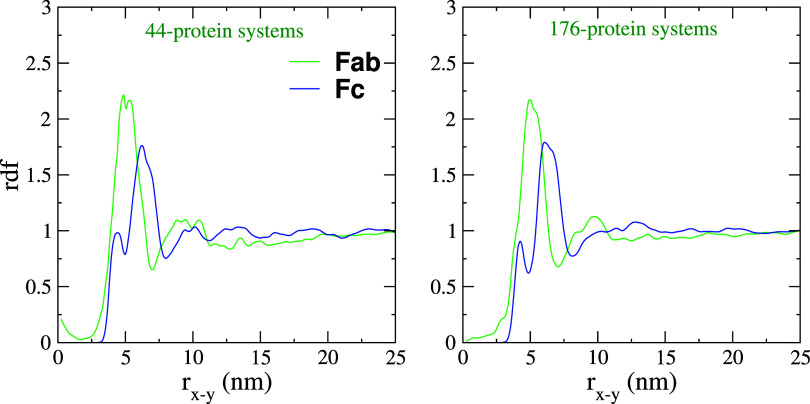
Radial distribution of the center of mass of the protein molecules projected on the interface plane (X–Y). The rdfs were averaged over three independent MD trajectories. The non-zero values at $r_{x-y}\to 0$ emerge from correlations of a reference protein with proteins located in the secondary layer that might have *x* and *y* coordinates close to those of the reference protein in the primary layer.

The protein monolayers' in-plane structure was quantified by calculating the in-plane radial distribution functions (*rdf*). We obtained the *rdf* by using the inter-protein center of mass distances. The *rdf*s show a clear main peak at about 5 nm (see Fig. 13), in agreement with the radius of gyration ( $R_{g}=2.5-2.6$ nm), computed in this work (see Figs. S1 and S2 in the supplementary material). The double peak (two peaks close to r_*x*−*y*_ = 5 nm) for the Fc systems appears because proteins are slightly out of the plane. The second lower peak (at r_*x*−*y*_ ∼5 nm) in the Fab and Fc structures provides evidence for protein-excluded volume effects, defining the short-range structure of the monolayer. However, the *rdf*s decay to 1 rather quickly, indicating the lack of a significant long-range order in the Fab or Fc protein monolayers.

### D. Modification of the protein structure and adsorption behavior due to oil–protein interaction

Experiments and simulations of protein adsorption at the oil-water interface involve the necessary interaction between the oil molecules and the proteins. It is unclear how these interactions modify the protein structure and inter-protein interaction. To shed light on the relevance of these interactions, we performed simulations of a single Fc/Fab fragment, initially placed at the oil–water interface, with half of the protein in contact with the water phase and the other half with the oil phase (see Fig. S11 in the supplementary material). We refer to this configuration as *Int.*, while configuration with the protein placed initially at the center of the water phase is referred to as *Cen.* The *Int.* simulations aimed to mimic experimental situations where the protein fragments may get dispersed in the oil phase due to vibrational stress during transport and storage.

**FIG. 14.**
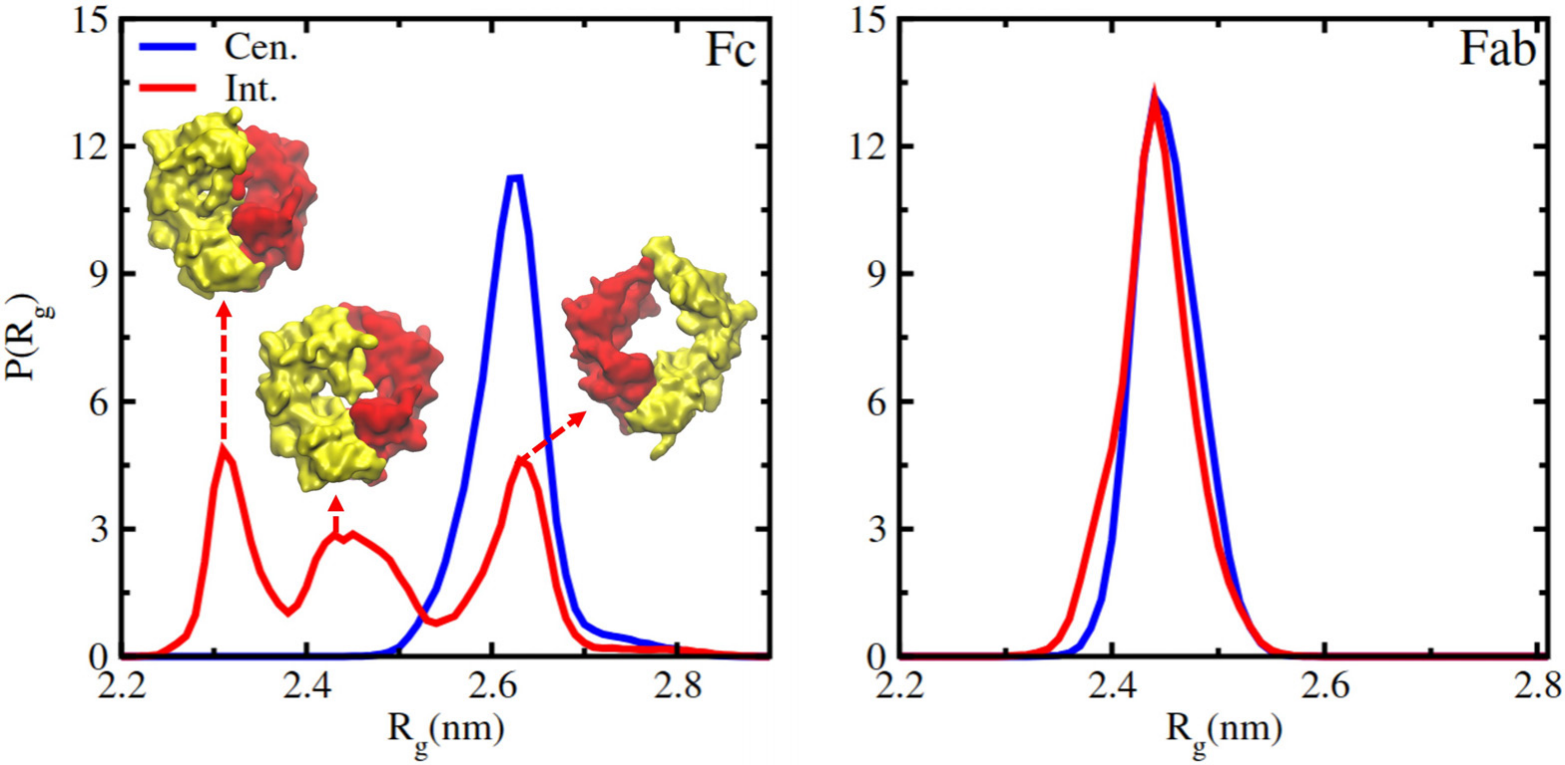
Probability distribution of the radius of gyration for simulations performed with the Fc or Fab proteins located initially at the center of the water phase (Cen.) or with the center of mass at the water–oil interface (Int.).

Our *Int.* simulations demonstrate that the oil molecules can induce (or not) significant structural distortions in the proteins. The *R_g_* of the Fab was found to be fairly insensitive to the oil–protein interaction (see Fig. 14). However, significant distortion is observed in the case of Fc. The analysis of 10 independent trajectories for this protein shows that the *R_g_* can adopt very different values (see Fig. 14 and Fig. S11 of the supplementary material), which result in a probability distribution with several maxima, revealing the formation of several competing conformations (see *R_g_* peaks at ∼2.3,2.45, and 2.6 nm in Fig. 14) that are much more compact as compared to the Fc fragment in bulk water and that in the *Cen.* simulation, indicating a highly distorted protein structure (see insets in Fig. 14).

To study the effect of the observed structural distortions of the Fc fragment on its adsorption behavior, we calculated the residue-wise A_*ads*_ for the Fc fragment simulated with the *Int.* setup. The A_*ads*_ surface color plot for the Fc surface is shown in Fig. S12 in the supplementary material. Fc adsorption proceeds through multiple regions on the protein surface, contrasting with the more localized surface active regions reported in the oil–water systems when the protein adsorbs from the aqueous phase in the *Cen.* simulation setup, which clearly showed adsorption through a localized region around the hinge (cf. Figs. 5 and S12 of the supplementary material).

Upon closer inspection of the *Int.* trajectories, we found that the oil molecules penetrate the protein structure (see Fig. S13 in the supplementary material), protruding from the protein surface into the water bulk solution. The absorption of oil molecules into the protein's hydrophobic core further contributes to the distortion of the protein structure. The Fc fragment, with higher flexibility, appears to be more prone to oil absorption than the Fab fragment. The absorbed oil molecules also contribute to adsorption through certain regions that do not pin to the interface in the *Cen.* simulations.

## III. DISCUSSION AND CONCLUSIONS

We have performed coarse-grained molecular dynamics simulations of the Fab and Fc fragments of mAb COE3 at the water–hexadecane interface by employing the coarse-grained model MARTINI 3. This force field is widely used in simulations of biomolecules and allows for significantly extended simulation time and length scales, compared to atomistic simulations. Using this model, we have investigated inter-protein and protein–water–oil–interface interactions using large-scale simulations.

In preparation for our interfacial simulations, we modified the original MARTINI3 model by changing the strength of the water–oil interaction to match the experimental water–hexadecane interfacial tension and rescaling the protein–water interaction to reproduce the experimental second virial coefficient (measured using SANS in our recent work^28^) of the Fc protein pairs. We showed that the rescaled protein–water interaction parameters reproduce the second virial coefficient of lysozyme, providing scope for our parameters to be used in other protein systems. The coarse-grained models enabled the investigation of the interaction of large protein assemblies, consisting of 10^2^ protein fragments, with the hexadecane–water interface, for extended times (up to 2 *μ*s). These simulations provide microscopic insight into the interaction of the proteins with fluid interfaces, the potential role of inter-protein interactions on co-adsorption, and the structure of the adsorbed protein layers. The latter complements recent experimental studies using neutron reflectivity experiments.

Simulations mimicking diluted and concentrated (targeting high surface concentrations, relevant in experiments ∼30 nmol/m^2^) protein solutions of the Fab or Fc fragments show that the Fab and Fc fragments adsorb at the oil–water interface through small regions, hot spots, on the protein surface containing amino acids like Tyr, Phe, Leu, Pro, Cys, and Ile. We quantified the protein adsorption at the water–oil interface by calculating the adsorbed area on a per-residue basis. The surface active amino acids in the hot spots do not change with protein concentration. However, the adsorbed area per amino acid increases with protein concentration. We interpret this result as a signature of synergistic adsorption, or co-adsorption, which emerges from inter-protein interactions when the in-plane inter-protein distance is ∼5 nm, corresponding approximately to the protein diameter (2 $R_{g}$).

Our microsecond long simulations using single and multi-protein systems show that Fc is more hydrophobic than Fab in the presence of the water–oil interface. This observation is consistent with the experimental analyses of adsorption, which revealed a faster change in water–hexadecane interfacial tension for the Fc fragment as compared to Fab.^23^ Analysis of the simulations shows that most (>70%) of the independent Fc (single protein) trajectories feature strong adsorption. In contrast, 30% of the Fab trajectories show intermittent adsorption. The adsorption of Fc is driven by the hydrophobic hinge region. The hinge sequence studied here is similar to various commercially available IgG1-based mAb therapeutics like trastuzumab and rituximab. Hence, we expect our results to apply to a broader range of therapeutic proteins.

The hot spots present in the Fc hinge region and near the N terminus of the V_*H*_ and V_*L*_ domains in the Fab fragments regulate protein adsorption at the water–oil interface and induce a preferred protein orientation with respect to the vector normal to the interface place. For Fab, the orientation angle is 46° ± 14°, 43° ± 20°, and 44 ± 19° for the single, 44- and 176-multi-protein systems, respectively. We found similar angles for the Fc fragment: 48° ± 11°, 44.6° ± 12°, and 45.3° ± 12°, respectively. These results show that the orientation angle is insensitive to the interfacial protein concentration.

The protein adsorption mediated by the hot spots and the preferred protein orientation relative to the interface plane define the effective protein monolayer thickness, 13.7 and 8.9 nm for Fab and Fc, respectively. These thicknesses are considerably larger than those obtained from slab models by fitting neutron reflectivity data. The larger thickness observed in our simulations is associated with a diffuse secondary protein layer. In the experimental analysis described in Ref. 35, the authors evaluated both single-slab and dual-slab models, with the latter providing a better fit with the neutron reflectivity data in most instances. This aligns with the existence of a more diffuse adsorbed layer as seen in our simulations. The differences in the total layer thicknesses obtained from the simulations and experiments primarily originate from different measurement methods or definitions of the parameters. In simulations, the thickness is determined by the distance of the furthest atom from the interface. In the slab models used for fitting of neutron reflectivity data, slabs are assumed to have uniform density. Therefore, at interfaces that are not sharply defined but instead show a gradual spread of molecular or atomic densities, the slab model averages these profiles, often resulting in an underestimation of thickness. Setting aside these differences, the simulation data are consistent with the experimental observations, particularly regarding the small penetration of the proteins into the oil phase.

We find that the “pinning” of the protein to the interface through the surface active regions results in a very small protrusion of the proteins into the oil phase, approximately 8% and 11% of the lengths of the long axes of the Fab and Fc fragments.^28^ This small protrusion is consistent with previous neutron reflectivity experiments.

We have found that protein–oil interactions do not significantly modify the conformation of Fab fragments. However, a strong interaction with oil (induced due to mechanical agitation) induces significant distortions in the Fc protein structure, translating into enhanced protein adsorption associated with an increase in surface active regions on the Fc surface. This contrasts with the surface active regions observed in proteins that adsorb from the aqueous phase and feature a weak interaction (through a limited region on the protein surface) with the oil phase. The simulation also demonstrates that hexadecane molecules can adsorb inside the Fc protein structure leading to further structural deformation.

The observation that the Fc fragments with distorted structures feature more surface active regions on the surface suggests that such deformed fragments might be more prone to aggregation. Therefore, it is possible that in practical setups where proteins, in the presence of a water–oil interface, are subjected to vibrational stress during storage and transport, the agitation could lead to the penetration of proteins into the oil phase, which, upon returning to the interface and eventually to the aqueous phase, could feature increased effective hydrophobicity, possibly impacting protein aggregation.

There are several possible extensions of our work. First, the analyses performed here could be extended to full antibodies. Such a study is needed to assess how the mAb structure modifies the adsorption hot spots on the protein surface, particularly near the hinge region. The analysis of antibody monolayers might open the route to investigating the mechanical properties of mAb monolayers, an important problem in surface rheology. Finally, the impact of oil molecules on protein conformation and aggregation will require additional work, particularly to understand how oil penetration and protein disruption modify inter-protein interaction.

## IV. METHODS

### A. Atomistic and coarse-grained modeling of the Fab and Fc fragments

We performed simulations of the Fab and Fc fragments of the monoclonal antibody COE3. The Fc domain of COE3 has the same sequence as the Fc domain of the human IgG B12, which can be found in the Protein Data Bank (1HZH).^31^ The Fab domain in COE3 shares a 73% similarity in sequence with the Fab domain in 1HZH. The atomistic model of the Fc fragment was obtained by removing the two Fab domains from 1HZH, and the Fab structure was taken from an earlier work by Singh *et al.*^32^

We aim to understand the interplay of local protein denaturation and protein adsorption. As mentioned in the introduction, previous experimental studies showed protein unfolding at fluid interfaces. Keeping this in mind, we created four different models of the Fc and Fab fragments using the martinize2 script.^33^ We first built the elastic network models^34^ of the proteins using the martinize2 script,^33^ wherein protein beads within a cutoff distance of 0.9 nm were connected with elastic springs (*k* = 500 kJ/mol). Two different kinds of elastic network models were built. In one of these models, we kept the hinge free of elastic bonds, while in the other model, we connected the beads belonging to the hinge region to nearby domains by elastic bonds based on the cutoff distance.

We also built MARTINI models of the proteins using the Go approach.^35^ The Go model replaces the harmonic bonds of the elastic network model with Lennard-Jones (LJ) interactions between relevant beads. The beads to be connected are selected based on their atomic overlap (OV).^36^ To check for atomic overlaps, each atomistic residue of the protein is defined as a collection of atomic spheres with radii equal to 1.25 times the vdW radii of the atoms. Additionally, the chemical nature of the overlapping atoms is considered to decide if two overlapping residues belonging to different residues should be connected by a Go bond (OV + rCSU).^35,37^ We defined the interaction strength of the LJ potential to be ε= 10 or 12 kJ/mol to build two different Go models for each protein. Unlike the elastic model, the Go model allows protein deformation, with breakable Lennard-Jones interactions replacing the unbreakable harmonic restraints. This would be useful for us in studying phenomena where the protein structure is deformed due to its environment, as discussed later in the paper.

Thus, in total, we built four different models of the Fab and Fc fragments for further evaluation.

#### 1. pH-dependent amino acid charges in MARTINI

To accurately model the pH-dependent charges of amino acids, we first determined the charge state of the amino acids at different pH conditions using propKa3.1.^38^ Specific His and Glu residues in the Fab and Fc fragments were protonated to account for positively charged His and neutral Glu residues. To convert the His residues from neutral to charged, the neutral MARTINI 3 TN5a bead in the histidine side chain was changed to a positively charged TQ2p bead. Whereas, protonated Glu residues were modeled by replacing the negatively charged Q5n side chain bead with a neutral MARTINI 3 P2 bead.

### B. Model evaluation

We used the four models described above to perform simulations in bulk water. The coarse-grained models, with amino acid charges corresponding to pH = 7 (leading to +2e charge for Fc and +11e charge for Fab), were placed in cubic periodic boxes of side 12 nm. The boxes were then solvated with MARTINI 3 non-polarizable water (no antifreeze particles) and the recommended value of 15 for the dielectric constant was used along with reaction field electrostatics. The systems were then neutralized by adding Cl^−^ ions (11 for Fab and 2 for Fc). Following charge neutralization, 148 Na^+^ and an equal number of Cl^−^ ions were added, corresponding to an added salt concentration of 150 mM.

All the systems underwent minimization using the steepest descent algorithm to eliminate unwanted overlaps between protein and water beads. After minimization, the system was equilibrated for 100 ns in the NVT ensemble at a temperature of 300 K. The system density was then equilibrated by performing a 200 ns long NPT simulation at a temperature of 300 K and a pressure of 1 bar. Three independent production runs (2 *μ*s each) were carried out for each system in the NPT ensemble. The temperature was maintained at 300 K using the v-rescale thermostat with a temperature coupling time of 2 ps. The Berendsen barostat was used during the NPT equilibration (pressure coupling time of 4.0 ps), and during production, the Parrinello–Rahman barostat with a coupling time of 12.0 ps was employed. All simulations used a vdW cutoff of 1.3 nm and reaction field electrostatics with the same cutoff. All simulations were performed using the GROMACS(2021.3) package.

The analysis of the four models described above provided information on how accurately each approach could reproduce the size of the protein, which was quantified by calculating the radius of gyration (R_*g*_). The models also provided insight into the protein adsorption mechanism. This is a particularly relevant aspect for the Fc domain since it is known to be more flexible than the Fab domain.^28^ Specifically, the elastic model leads to more rigid protein structures than the Go model, which allows a certain degree of protein deformation with respect to the initial structure. The differences in the protein flexibility predicted by the elastic and Go models might lead to different preferences for the exposure of hydrophobic residues at the protein surface, ultimately influencing the protein surface activity.

Figure S1 in the supplementary material shows the probability distribution of the R_*g*_ of the Fc domain for the different models investigated in this work. The elastic network models underestimate the R*_g_* relative to the all-atom and Go models, suggesting that elastic bonds lead to more compact protein structures. The Go model predicts slightly larger *R_g_* (2.64 nm) than the all-atom model (2.6 nm). We do not find a strong dependence of *R_g_* with the *ε* LJ parameter of the Go model. The average *R_g_* predicted by the Go model is in good agreement with the experimental measurements (SANS) at pH = 7 and 300 K (2.64 nm).^28^

In Fig. S2 of the supplementary material, we compare the probability distribution of *R_g_* for the Fab fragment, obtained using the Go model, against the R_*g*_ of Fab modeled using the all-atom force field Charmm36m *ff*. For the interaction parameter ε=10 kJ/mol, we obtain an average *R_g_* (2.5 nm) in reasonable agreement with the all-atom computations (2.52 nm). We show in Fig. S2 that the average *R_g_* is fairly sensitive to the chosen *ε*, and for 12 kJ/mol, we obtain a significant deviation from the all-atom results. At pH = 7 and 300 K, the *R_g_* obtained from SANS measurements on the Fab fragment in water is 2.5 nm.^28^ Overall, the *R_g_* obtained with Go models ( ε=10 kJ/mol) shows reasonable agreement with the experimental values. We use the Go model with ε=10 kJ/mol for all the simulations discussed below.

### C. Potential of mean force calculation and second virial coefficients

Previous studies have shown that the MARTINI 3 force field overestimates protein compactness and inter-protein attractive interaction compared to experimental results.^26,27^ To better understand the nature and strength of the inter-protein interaction and measure the ability of the MARTINI 3 model to reproduce experimental behavior, we calculated the potential of mean force (PMF) between two Fc proteins using the umbrella sampling technique.^39^ The PMF profiles, *W*(*r*), were used to determine the second virial coefficient,

$$B_{22}=-\frac{2\pi N_{A}}{M_{w}^{2}}\int_{0}^{\infty }(g(r)-1)r^{2}dr,$$ (2)

where *M_w_* is the molecular weight, 49 635 g/mol,^23^ of the Fc fragment and *r* is the inter-protein distance. Equation (1) assumes the protein has a spherical shape. We approximate the pair radial distribution function, *g*(*r*), in the integrand as follows:

$$g(r)=\text{exp}\left(\frac{-W(r)}{RT}\right),$$ (3)

We conducted simulations at a constant pressure of 1 bar, and at a temperature of 300 K. *W*(*r*), thus, represents the Gibbs free energy. To compute the PMF, we used eight different starting configurations for two Fc domains in water. These configurations had different initial relative orientations of the two Fc fragments (see Fig. S3 of the supplementary material). We placed the Fc fragments in a box of dimension 18 × 18 × 18 nm^3^, solvated in non-polarizable MARTINI 3 water, and neutralized by adding 4 Cl^−^ ions, since the charge of Fc at pH = 7 is +2e. We added 540 Na^+^ and Cl^−^ ion pairs to achieve a salt concentration of 150 mM. The systems were minimized using the steepest descent method to remove bad contacts between the different beads. A 200 ns long equilibration run was performed in the NPT ensemble using the v-rescale^40^ thermostat (temperature coupling constant of 1 ps) and the Berendsen barostat^41^ (pressure coupling constant of 4 ps). After equilibration, we pulled the two Fc domains apart and extracted conformations from the pulling trajectory, which were selected as starting configurations for umbrella sampling windows. We simulated 57 windows, with the Fc–Fc distances varying from 2.9 to 8.5 nm with a gap of 0.1 nm between two consecutive windows. For each window, we performed a 200 ns long equilibration using the v-rescale thermostat (temperature coupling constant of 1 ps) and the Berendsen barostat^41^ (pressure coupling constant of 4 ps). Following the equilibration, a 2 *μ*s long production run was performed in the NPT ensemble using the v-rescale^40^ thermostat (temperature coupling constant of 1 ps) and the Parrinello–Rahman barostat^42^ (pressure coupling constant of 12 ps). The trajectory generated during the production run was used for PMF calculation using the weighted histogram analysis method^43^ (WHAM). The error in *W*(*r*) was calculated using bootstrapping (200 bootstraps were used). The errors reported for the virial coefficients were obtained from an error propagation applied to Eq. (1).

### D. Simulation of the Fc/Fab domains at the water–hexadecane interface and characterization of protein adsorption

#### 1. Simulations of proteins at the oil–water interface

We adjusted the protein charges to a value consistent with pH = 5.5. Using the propKa analysis, we found the charge of the Fc fragment to be +9e and that of the Fab fragment to be +17e. The locations of amino acids protonated at pH = 5.5 for the Fab and Fc fragments are shown in Fig. S4 of the supplementary material. To build the system with a single Fc/Fab domain at the water–oil interface, we placed the protein center of mass at (7.5 nm, 7.5 nm, 7.5 nm) inside a simulation box with dimensions 15 × 15 × 30 nm^3^. Half of the box, up to 15 nm, along the Z-direction was solvated with water, and the other half contained hexadecane (see Fig. 1). Therefore, the protein was positioned in the middle of the water slab. 51 Na^+^ and 60 (68) Cl^−^ ions were added to the water phase for the Fc (Fab) systems to neutralize the charge and reach a salt concentration of 25 mM. The pH and salt concentration used in our work is the same as in earlier experimental work performed for the same system.^23^

**FIG. 15.**
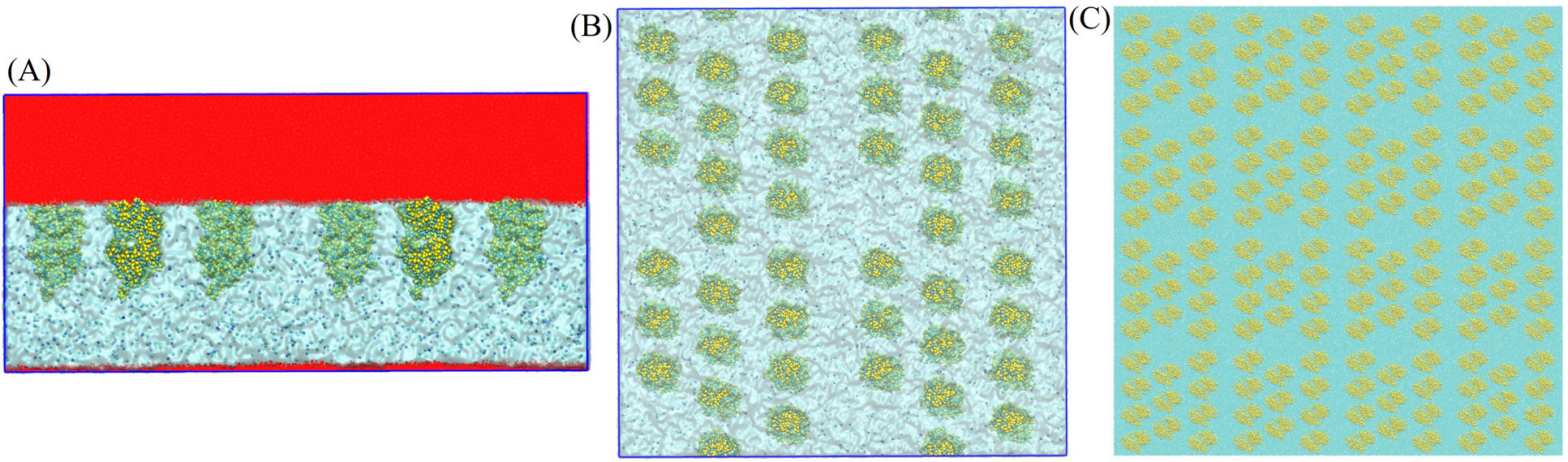
Initial systems for the multiple-protein simulations. (a) Side view of the initial systm containing 44 Fab proteins: the hexadecane region is shown in red, water and ions are shown in cyan, and the proteins are shown in a bead representation. (b) Top view (hexadecane not shown) of the initial system containing 44 Fab proteins. (c) Top view of the 176-Fc protein system.

We also performed simulations of multiple Fab/Fc fragments at the water–oil interface. Two types of multi-protein systems were built (see Fig. 15), one containing 44 Fab/Fc fragments and a larger system with 176 Fab/Fc fragments. In the multiple-protein simulations, the Fab/Fc fragments were initially placed at the interface, pinning the oil phase. The orientation of the Fab/Fc fragments in the multi-protein systems was set to the most probable orientation of the proteins obtained from the single-protein simulations. We checked that the proteins detached and readsorbed from/to the oil–water interface, hence ensuring the system reaches the equilibrium state (see Fig. 4). The cross section (X–Y) of the box for the 44-protein system was 25 × 25 nm^2^, while that for the 176-protein system was 100 × 100 nm^2^. The initial number of proteins used corresponded to an interfacial density of 30 nmol/m^2^, which is similar to the density inferred from the analyses of neutron reflectivity experiments performed with a bulk protein density of ∼100 ppm.^23^ While the initial orientation was taken from the single-protein simulation, the interfacial density of 30 nmol/m^2^ resulted in the proteins having enough space to reorient, as is clear from Fig. 15. The initial length of the simulation box in the direction perpendicular to the interface plane (Z) was 25 nm. Water was added in the region defined by *Z* = 0–15 nm, and the remainder of the simulation cell was filled with hexadecane.

After placing the proteins at the interface, 748 (396) Cl^−^ ions were added for the 44-protein Fab (Fc) system, and an additional 548 Na^+^ and Cl^−^ ions were added to maintain a salt concentration of 25 mM. For the 176-protein construct, 2992 (1584) Cl^−^ ions were added to neutralize the Fab (Fc) system, and an additional 2192 Na^+^ and Cl^−^ ions were added, again to obtain a salt concentration of 25 mM.

#### 2. Protocol for interface simulations

The oil–water–protein systems discussed above were minimized using the steepest decent algorithm and equilibrated for 100 ns in the NVT ensemble (300 K). After that, all the systems were equilibrated in the NPT (300 K, 1 bar) ensemble using semi-isotropic pressure coupling. During this process, the X–Y dimensions of the box were kept fixed, and the Z-dimension was allowed to change. Following the equilibration, we performed three independent production runs of 2 *μ*s for each system in the NPT ensemble. To maintain the temperature at 300 K, we used the v-rescale thermostat with a temperature coupling time of 2 ps. An integration time step of 20 fs was employed. During equilibration, we used the Berendsen barostat with a pressure coupling time of 4.0 ps, while during the production runs, we employed the Parrinello–Rahman barostat with a coupling time of 12.0 ps. These simulations used a vdW cutoff of 1.3 nm and reaction field electrostatics with the same cutoff. We performed ten independent simulations for the single-protein systems and three separate runs for the multi-protein systems. All simulations were performed using the GROMACS 2021.3 package.

We also performed simulations of the water–oil interface without proteins. We computed the oil–water interfacial tension using the pressure tensor route, $\gamma =\frac{L_{z}}{2}\left(P_{zz}-\frac{1}{2}\left(P_{xx}+P_{yy}\right)\right)$, where $P_{\alpha \alpha }$ are the diagonal components of the pressure tensor, and the coordinate *z* is normal to the interface plane. The interfacial tension (*γ*) for the water–hexadecane interface obtained from these simulations was 44.5 mN/m, which is lower than the experimental value.^23–25^ To match the experimental interfacial tension, we systematically reduced the vdW interaction energy (*ε*) for the water–oil interaction from its original value in MARTINI 3, 2.07 kJ/mol, to 1 kJ/mol, and calculated *γ* for each value of *ε*. In this way, we identified the optimal *ε* needed to reproduce the experimental oil–water interfacial tension. This value of *ε* was used in all the interfacial simulations described above. Further details are presented in Secs. II and III. The simulation protocol used for oil-water simulations was the same as described above for the simulation with proteins.

#### 3. Determining surface active regions

To determine the surface active regions of the Fab and Fc fragments, we calculated the area of each amino acid residue at the water–oil interface. We used the Gromacs code gmx sasa with the -or, -surface (input: System), and -output (input: Protein) flags. Without the -or flag, one obtains the total area of the protein that protruded into the oil phase (A_*ads*_). We used a probe radius of 0.27 nm to calculate the area.

A non-zero average value of the adsorbed area (A_*ads*_) was taken as an indicator of protein adsorption. We used the time-averaged residue-wise A_*ads*_ to create color maps projected on the corresponding amino acids at the protein surface. This allowed us to highlight surface active regions. Further discussion of this topic is presented in Sec. II.

## SUPPLEMENTARY MATERIAL

See the supplementary material for details on the structural properties of the Fc fragment using the elastic network and Go models, a representation of the Fab and Fc surface, PMF and B_22_ data for Fc–Fc and lysozyme, additional protein orientation and density profiles, protein adsorption trajectories, and a simulation snapshot demonstrating the interaction of oil molecules with the Fc fragment.

## Data Availability

The data that support the findings of this study are available within the article and its supplementary material.
